# Comparison of the effects of burn assessment mission game with feedback lecture on nursing students’ knowledge and skills in the burn patients’ assessment: a randomized clinical trial

**DOI:** 10.1186/s12911-024-02558-4

**Published:** 2024-06-05

**Authors:** Amirreza Nasirzade, Kolsoum Deldar, Razieh Froutan, Mohammad Taghi Shakeri

**Affiliations:** 1 Department of Medical Surgical Nursing, School of Nursing and Midwifery, Mashhad University of Medical Sciences, Mashhad, Iran; 2 Department of Information Technology, School of Allied Medical Sciences, Shahroud University of Medical Sciences, Shahroud, Iran; 3 Nursing and Midwifery Care Research Center, Mashhad University of Medical Sciences, Mashhad, Iran; 4 Department of Epidemiology and Biostatistics, School of Health, Mashhad University of Medical Sciences, Mashhad, Iran

**Keywords:** Serious game, Feedback lecture, Nursing students, Burn assessment, Randomized controlled trial

## Abstract

**Background:**

Learning of burn patient assessment is very important, but heart-breaking for nursing students. This study aimed to compare the effects of feedback lecture method with a serious game (BAM Game) on nursing students’ knowledge and skills in the assessment of burn patients.

**Method:**

In this randomized controlled clinical trial, 42 nursing students in their 5th semester at Mashhad University of Medical Sciences School of Nursing and Midwifery, were randomly assigned to intervention (BAM game, available for two weeks) and control (feedback lecture method presented in two 90-minute sessions) groups. Two weeks after the intervention, all students were evaluated for their knowledge (using knowledge assessment test) and skills (using an Objective Structured Clinical Examination). Statistical analysis involved independent t-test, Fisher’s exact test, analysis of covariance (ANCOVA), and univariable and multivariable ordinal logistic regression models.

**Results:**

Following the intervention, the skill scores were 16.4 (SD 2.2) for the intervention group and 11.8 (SD 3.8) for the control group. Similarly, the knowledge scores were 17.4 (SD 2.2) for the intervention group and 14.7 (SD 2.6) for the control group. Both differences were statistically significant (*P* < .001). These differences remained significant even after adjusting for various factors such as age, gender, marital status, residence, university entrance exam rank, and annual GPA (*P* < .05). Furthermore, the BAM game group showed significantly higher skills rank than the feedback lecture group across most stations (eight of ten) (*P* < .05) in the univariable analysis. Multivariable analysis also revealed a significantly higher skills score across most stations even after adjusting for the mentioned factors (*P* < .05). These results suggest that the BAM game group had higher skills scores over a range of 1.5 to 3.9 compared to the feedback lecture group.

**Conclusions:**

This study demonstrated that nursing students who participated in the BAM game group exhibited superior performance in knowledge acquisition and skill development, compared to those in the control group. These results underscore a significant enhancement in educational outcomes for students involved with the BAM game, confirming its utility as a potent and effective pedagogical instrument within the realm of nursing education.

**Trial registration:**

Iranian Registry of Clinical Trials: IRCT20220410054483N1, Registration date: 18/04/2022.

**Supplementary Information:**

The online version contains supplementary material available at 10.1186/s12911-024-02558-4.

## Introduction

Burn patients experience physical, mental, and psychological complications in cases of incorrect and late assessment, which imposes an extremely heavy economic burden on them [1, 2] and worsens the life-threatening complications caused by burns. If competent nurses assess burn victims early, they can prevent many burn complications and emergencies, such as circulatory disorders, airway damage, and compartment syndrome, and save the patients’ lives [3–5]. On the other hand, such emergencies need capable and skilful nurses to be prevented [6]. These skills and competencies depend on improving one’s knowledge and cognition [7].

Lecturers and professors continually challenge to develop teaching-learning methods with appropriate content and structure that can effectively enhance nursing students’ assessment knowledge and skills, allowing them to access these skills anytime and anywhere [8–10]. One of the primary concerns of universities worldwide is to enhance the professional competency of students and establish a strong link between theoretical knowledge and specialized skills [11]. Currently, nursing students acquire the assessment knowledge and skills related to patient through traditional methods, including lectures [12]. Among different lecture methods, the feedback lecture can lead to more involvement of students with different learning characteristics [13]. Although feedback lectures encourage critical thinking and problem-solving skills [14], they offer a limited number of repetitions for understanding educational materials and require prolonged follow-up time to tailor learning concepts [15]. Due to the challenge in translating written knowledge from reference books into clinical skills, many lecturers are turning to new technologies and approaches [16, 17] to strengthen decision-making processes and clinical reasoning [18].

Serious games in medical education employ game elements with goals that extend beyond mere entertainment [19]. Game-based learning boosts an individual’s motivation and engagement in learning the desired concepts and makes the learning experience enjoyable, convenient, and effective [20, 21]. A serious game simulates real-world events or processes aiming to educate users and has shown better outcomes than traditional classroom learning [22]. Serious games are beneficial to train health professionals and patients [23–26], an anaesthesia techniques [27], surgical procedures [28], and in the principles of cardiopulmonary resuscitation [29].

Serious games can elevate learners’ motivation and improve the efficacy of the learning environment. As a result, they have become a vital component of educational programs in universities. In well-designed serious games, users feel as though they are actively participating in and learning from a real-world experience [30]. Game-based learning environments provide individuals the chance to make mistakes without fear of serious consequences [31].

Sometimes, the educational content for medical students contains distressing and unpleasant materials that lead them to disengage from these materials and avoid memorizing them [32, 33]. For instance, students often show reluctance in engaging with the burn course due to distressing scenes and severe injuries [34, 35]. Moreover, the lack of clinical experience can significantly hinder nursing students’ ability to develop essential assessment skills [36]. Developing professional competence in health-related fields is a top priority for universities globally. This necessitates nurturing a close connection between theoretical knowledge and practical skills [11]. It appears that serious games, as a novel and appealing teaching method, could mitigate such challenges. This approach has been deemed more effective for acquiring knowledge and cognitive skills [37].

The creation of an educational board game for teaching burn care has been shown to enhance the knowledge of healthcare members [38], but, to the best of our knowledge, no study has yet explored the assessment of burn patients. Training nursing students in patient assessment skills is crucial to avoid fatal complications of burns. This study aimed to compare the impact of the feedback lecture method and a newly designed serious game on nursing students’ knowledge and skills in assessing burn patients.

## Method

### Study type and participants

In this randomized controlled clinical trial, all 5th-semester nursing students at Mashhad University of Medical Sciences (MUMS), School of Nursing and Midwifery (*n* = 44), were eligible to participate in the study. They were randomly assigned to intervention and control groups. The randomization process was carried out by an external third party, using random number lists obtained from an online randomization website (www.randomization.com). An assistance from a statistical consultant (blinded) was sought to randomly allocate the participants. Since all eligible students were included in the study, a sample size calculation was not performed.

### Inclusion and exclusion criteria

Inclusion criteria were undergraduate nursing students studying at School of Nursing and Midwifery of the MUMS, who had access to the Internet through a mobile phone or computer and were assessing burn patients for the first time, meaning they had no previous knowledge or skill about this field. According to the nursing curriculum in the MUMS, this course is offered in the 5th semester of nursing, and in the previous semesters, students had no theoretical knowledge or practical encounters with burn patients in the classrooms, workshops, or hospitals.

Exclusion criteria included students who were unwilling to continue the study, students in the control group who were absent in more than one session, and students in the BAM game (Burn Assessment Mission game) group who were absent for a third of the defined time.

### Blindness

This study employed a single-blind design, meaning the statistical counsultant was unaware of the subjects’ allocation to the intervention or control groups.

### Procedure

Preparation of educational content involved extracting the required material from valid burn references [39, 40] and then localizing it based on experts’ opinions.

#### Design of BAM game

The development of a serious game was inspired by the challenges encountered in teaching fifth-semester nursing students how to assess burn patients. The assessment scenes were unpleasant, and the clinical conditions were not conducive to effective teaching or active learning. Consequently, burn specialists, nursing professors, health informatics specialists, and a software engineering team conducted several brainstorming sessions to design the BAM game to address this challenge. In the BAM game, students could initially study educational content before real clinical encounter. Here, they would become familiar with crucial and vital points in assessing burn patients through real images and videos, thereby solidifying their learning. This method ensured that students could interact with the material in a dynamic and engaging manner, thereby enhancing the effectiveness and enjoyment of the learning process.

The first step involved preparing a comprehensive educational package based on the university’s educational protocol, created by the research team’s professors. Next, multiple educational scenarios were written based on the most common, real cases referred to the burn department. Relevant videos and images were also prepared for the BAM game design phase.

Then, the engineering team designed the BAM game using PHP language. The user interface was built using HTML/CSS and JavaScript technology, the processing of the submitted data was carried out by the PHP programming language, and MySQL was used to manage the data storage. Students could enter the BAM game after registering and setting their usernames and passwords. Initially, they were explained the importance of assessing burn patients and the rationale behind the game’s design. Then, students could view the guide and rules to play the game, as well as the educational content in the form of multimedia (Multimedia Appendix 1: “A quick review of the BAM game”).

The titles of the stages included assessments of circumferential burn injuries (limbs, chest, and abdomen), electrical burn, thermal burn (head and face), chemical burn, carbon monoxide poisoning, inhalation burn, delayed burn, and the extent and depth of burn (Table 1).

Questions related to each stage were presented in the forms of short films or images of real patients with burns, and students had to answer the questions within a certain time limit. Those who chose the correct option were encouraged, and their learning was confirmed. However, those who chose the wrong answer received a message stating that their choice was wrong, without being referred to the correct option, and it was explained why their answer was incorrect. Students could pass the current stage if they answered 60% of the questions. They could also compare their scores with other students and see their ranks in the classroom at any time. The game featured characteristics such as graphic elements, light colors, various emoji symbols indicating happiness and sadness, encouraging sound effects, stars and medals, an attractive appearance, and a sense of competition and excitement.

### Intervention group

Students in the intervention group learned the educational content through the BAM serious game and could use the game for two weeks. Every week, a reminder message (Short message / SMS) was sent to them to encourage participation in the game. The BAM game could calculate how many times and how long each person used the game, so if students were absent for one third of the required time, they were excluded from the study.

### Control group

Students in the control group received educational content related to the assessment of burn patients through feedback lectures within two 90-minute sessions; each 90 min were divided into three parts of 30 min. The lecturer spoke about the topic for the first 25 min and then answered students’ questions for five minutes; she used related pictures or videos depending on the educational conditions and content. In total, three 25-minute lectures with three 5-minute active discussions were held in each session. These sessions started prior to the initiation of the intervention for the BAM group to prevent contamination. Also, all participants signed confidentiality agreements about the importance of not sharing information about the sessions with peers from the other group. Finally, we conducted regular check-ins and monitoring of both groups to ensure adherence to the study protocols. This included brief interviews or surveys to detect if any sharing of information has occurred. Furthermore, the access to the software was restricted to each student via a uniquely defined code. This ensured that students without this code couldn’t enter the BAM, even if they had the software installation file.

### Outcome measurement

Our participants had no prior theoretical knowledge or clinical encounter in the filed of burn assessment and manegment. So there was no need for conducting a pre-test.

Two weeks after completing the educational course, all students were evaluated in two stages: (I) participation in the knowledge assessment test (30 min); and (II) objective structured clinical examination (OSCE) for skills assessment.

To measure the knowledge of all students, 20 multiple-choice questions were used. The cutoff points were selected as follows: “Low level (0–34%)”, “Moderate level (35%-69%)”, and “High level (> 70%)”. Scores for the low level range from 0 to 7, scores for the moderate level range from 7.1 to 14, and scores for the high level range from 14.1 to 20. Higher scores indicate greater knowledge. A score of 14 (70%) or higher indicates a sufficient understanding of burn patient assessment. A passing score was set at 10.

To evaluate the skills assessment, a checklist was prepared by the nursing professors of our team, assessing the students’ skills in various dimensions of burn assessment using the OSCE approach. This exam included 10 stations (scenarios) related to burn injuries of the limbs, chest and abdomen, head and face, carbon monoxide poisoning and inhalation injuries, chemical and electrical burns, delayed burns, and assessment of burn extent and depth, according to the syllabus. Each station received a numerical score ranging from 1 to 5. For the purpose of simplifying calculations, these scores were then scaled to a maximum of 20. The final score for each student was calculated by adding up the scores of all 10 stations and then dividing by 10. The cutoff points were the same as those for the knowledge questionnaire, described previously. The students had to pass each station within five minutes. A passing score for each station was set at 10.

### Reliability and Validity

The face and content validity, comprehensiveness, clarity and difficulty of the knowledge questionnaire was assessed, and after some modifications were confirmed by expert opinions (seven professors in the field of burn management). This scale demonstrated a satisfactory level of content validity, with a content validity ratio (CVR) of ranging from 0.72 to 0.91 and a scale content validity index (S-CVI) of 0.87 (ranging from 0.82 to 0.97), and face validity with a mean impact score of 2.35 (ranging from 2.24 to 4.45). The reliability of the calculation, as measured by the Kuder-Richardson Formula 20 (KR-20), was 0.7.

The content validity of the OSCE checklist was checked by expert consensus and iterative review and revision. The reliability of the skill assessment checklist items was confirmed using the inter-rater reliability method (ICC = 0.86), too.

### Statistical Analyses

Statistical analysis was conducted using appropriate methods and IBM SPSS Statistics software [ver.28] (IBM SPSS Statistics, Armlonk, NY, USA). Normality of the numeric variables was checked and confirmed by Kolmogorov- Smirnov test. Data were presented using mean (SD) or median (percentile 25 – percentile75) for the Numeric Normal and non-normal variables, respectively and frequency (percent) for categorical variables. The between group comparisons of baseline measures and demographic variables were carried out by independent t tests, Mann-Whitney tests, and Fisher-Freeman-Halton Exact tests where appropriate. The correlations among the main variables were measured using Pearson correlation test. To assess the effect of intervention on knowledge and skills total score, the analysis of covariance (ANCOVA) was used after controlling for covariates (including age, gender, marital status, residence, university entrance exam rank, and annual grade point average or GPA). The intervention on skills score across stations, was assessed using univariable and multivariable ordinal logistic regression models. In the multivariable analyses, the effect of some covariates (including age, gender, marital status, residence, university entrance exam rank and annual GPA) were adjusted. All analyses were carried out using intention to treat approach, and P values less than 0.05 considered as significant.

## Results

### Participants’ profile

**Fig. 1:**
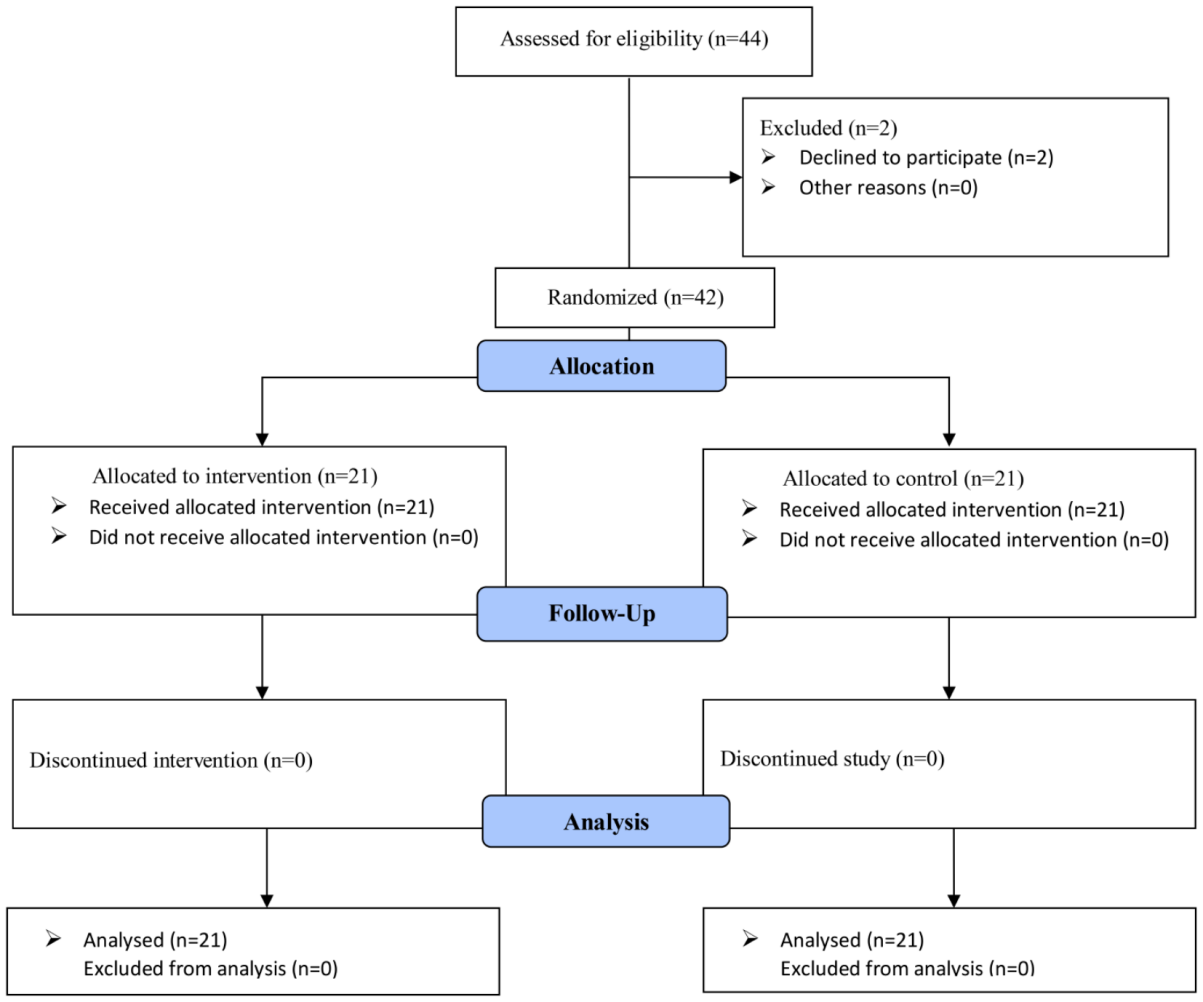
CONSORT flow diagram

Forty-four participants recruited in this study. In the first step evaluation for eligibility, two students were excluded (declined to participate). Finally, 42 patients were analyzed in the intervention (*n* = 21) and control (*n* = 21) groups (Fig. 1).

No significant difference was observed in terms of age, gender, marital status, residence, university entrance exam rank and annual GPA between intervention and control groups (Table 1).

**Table 1:** Participants’ profile

Variables	BAM Game		Feedback lecture		*P*-Value
	Mean/*n*	SD/(P25-P75)/%	Mean/*n*	SD/(P25-P75)/%	
Age (years)	21.2	1.1	21.6	1.0	0.159^T^*
Gender					0.335^F^**
Male	9	42.9	12	57.1	
Female	12	57.1	9	42.9	
Marital status (Married)	4	19.0	3	14.3	1.000^F^
Residence					0.753^F^
Dormitory	8	38.1	9	42.9	
Non-dormitory	13	61.9	12	57.1	
Annual GPA	17.3	0.6	17.6	1.1	0.235^T^
University entrance exam rank	3472/0	1135	3765.4	1246.6	0.43^T^

Med: median; n: number; GPA: grade point average

*T: independent t-test; **F: Fisher’s exact test

Significant differences were observed between intervention and control groups in terms of skills total score (*P* < .05), and knowledge score (*P* < .05). The differences remained significant after adjusting for of age, gender, marital status, residence, university entrance exam rank and annual GPA) both (*P* < .05) (Table 2).

**Table 2:** Comparing skills and knowledge scores between intervention and control groups

	BAM game		Feedback lecture		*P*-value*	*P*-value**
	Mean	Std. Deviation	Mean	Std. Deviation		
Skills total score	16.4	2.2	11.8	3.8	< 0.001	< 0.001
Knowledge score	17.4	2.2	14.7	2.6	< 0.001	< 0.001

* independent t-test

** Analysis of covariance, after adjusting for of age, gender, marital status, residence, university entrance exam rank, and annual GPA

### Correlations among main outcomes

The results showed that significant and positive correlations were observed between knowledge score and skills total score (*r* = .91, *P* < .05), the more the knowledge score the more the skills total score.

### Results of univariable and multivariable ordinal logistic regressions comparing intervention and control groups

**Fig. 2:**
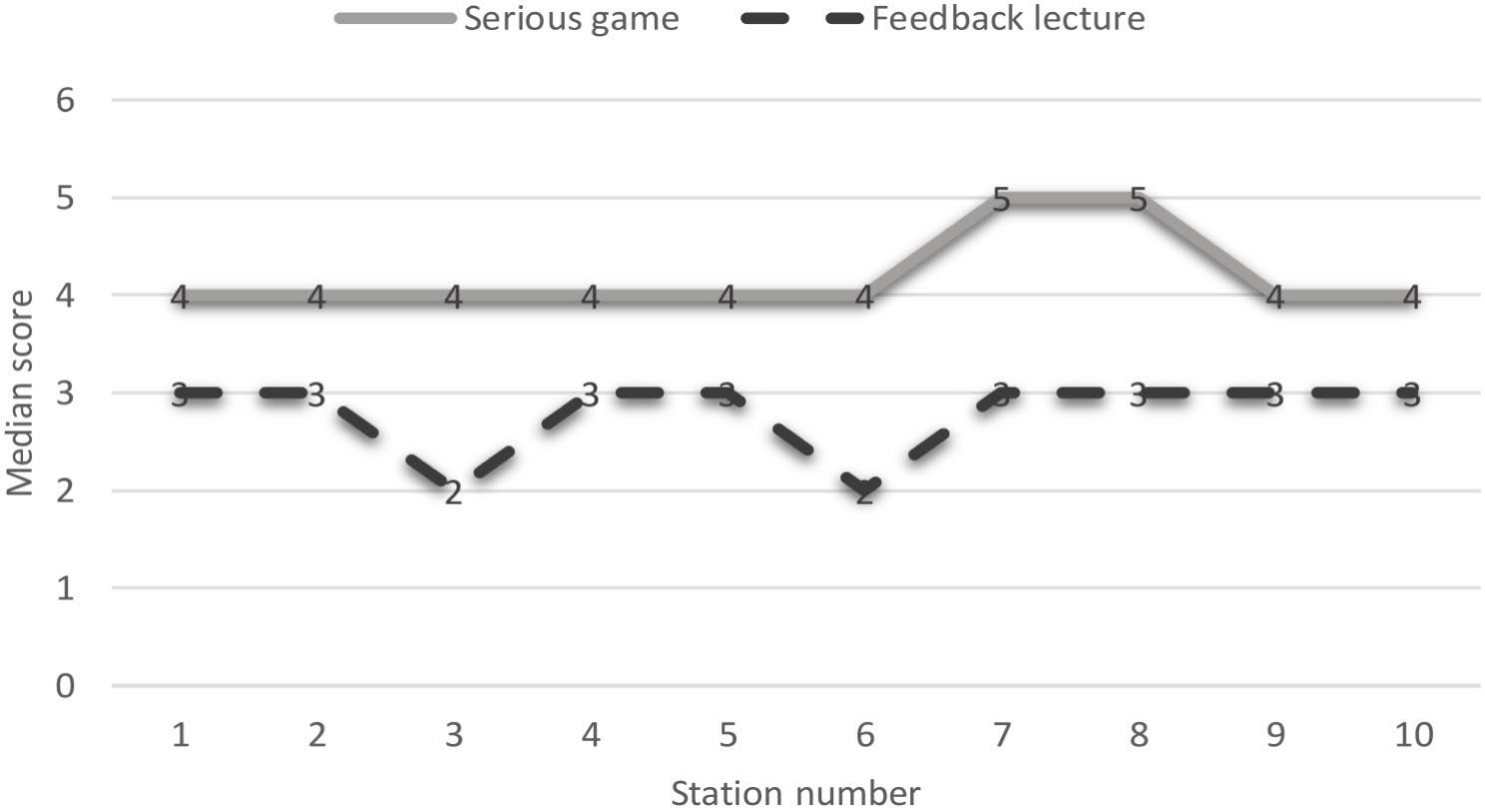
Median scores of skills in 10 station across intervention and control groups

The results univariable ordinal logistic regressions showed that for station 1 to station 8, the BAM game group had significantly higher skills rank compared to the feedback lecture group (all *P* < .05) (Table 3, and Table S1). So that, the BAM game group had higher skills score over a range of 1.6 to 3.9. Besides, after adjusting for of age, gender, marital status, residence, university entrance exam rank, and annual GPA, the results of multivariable ordinal logistic regressions indicated a significantly higher skills score across all stations (all *P* < .05), so that the BAM game group had higher skills score over a range of 1.5 to 3.9 (Table 3; Fig. 2).

**Table 3:** Results of univariable and multivariable ordinal logistic regressions comparing intervention and control groups

		Univariable analysis*				Multivariable analysis**				
		B	95% CI L	95% CI U	*P*-value	B	95% CI L	95% CI U	*P*-value	
Station 1 (Circumferential burns of the limbs)	BAM game	1.6	0.4	2.8	0.010	3.2	1.5	4.8	< 0.001	
	Feedback lecture	Ref.	.	.	.	Ref.	.	.	.	
Station 2 (Circumferential burns of the chest and abdomen)	BAM game	2.8	1.4	4.2	< 0.001	3.5	1.9	5.2	< 0.001	
	Feedback lecture	Ref.	.	.	.	Ref.	.	.	.	
Station 3 (electrical burn)	BAM game	3.9	2.1	5.6	< 0.001	4.8	2.8	6.7	< 0.001	
	Feedback lecture	Ref.	.	.	.	Ref.	.	.	.	
Station 4 (thermal burn)	BAM game	1.8	0.6	3.1	0.004	3.0	1.4	4.5	< 0.001	
	Feedback lecture	Ref.	.	.	.	Ref.	.	.	.	
Station 5 (chemical burn)	BAM game	1.6	0.4	2.8	0.007	2.7	1.2	4.1	< 0.001	
	Feedback lecture	Ref.	.	.	.	Ref.	.	.	.	
Station 6 (CO poisoning)	BAM game	2.2	0.9	3.5	< 0.001	3.3	1.7	4.9	< 0.001	
	Feedback lecture	Ref.	.	.	.	Ref.	.	.	.	
Station 7 (inhalation burn)	BAM game	3.2	1.7	4.7	< 0.001	3.9	2.2	5.6	< 0.001	
	Feedback lecture	Ref.	.	.	.	Ref.	.	.	.	
Station 8 (delayed burn)	BAM game	2.3	1.0	3.7	< 0.001	3.9	2.0	5.9	< 0.001	
	Feedback lecture	Ref.	.	.	.	Ref.	.	.	.	
Station 9 (extent of burn)	BAM game	0.9	-0.3	2.0	0.135	2.0	0.5	3.5	0.011	
	Feedback lecture	Ref.	.	.	.	Ref.	.	.	.	
Station 10 (depth of burn)	BAM game	0.8	-0.4	1.9	0.184	1.5	0.1	2.8	0.034	
	Feedback lecture	Ref.	.	.	.	Ref.			.	

CI: confidence interval; L: lower bound; U: Upper bound; CO: carbon monoxide

* Univariable ordinal logistic regression model to compare groups

** Multivariable ordinal logistic regression model to compare groups, after adjusting for of age, gender, marital status, residence, university entrance exam rank, and annual GPA

## Discussion

This study compared the effectiveness of serious game-based learning with feedback lectures in enhancing students’ knowledge and skills in assessing burn patients. The intervention and control groups were initially similar in key demographic characteristics. The results showed that students who participated in the serious game-based learning method significantly improved their assessment knowledge and skills compared to those who received feedback lectures. The analysis using univariable ordinal logistic regressions compared the two educational approaches across eight stations. It revealed that participants in the BAM game group exhibited significantly higher skill levels in eight out of ten stations compared to the feedback lecture group. The multivariable ordinal logistic regression analysis, which accounted for age, gender, marital status, place of residence, university entrance exam ranking, and annual GPA, confirmed these findings. Both analyses showed that the BAM game-based teaching method significantly enhances skill levels compared to feedback lectures, with this advantage remaining significant across various evaluation settings, even after adjusting for demographic and academic variables.

The study results indicated that serious games improved burn patient assessment knowledge and skills compared to the feedback lecture in nursing students. The majority of research highlights the effectiveness of education through serious games. For example, Farsi et al. (2021) reported the positive effect of serious games on the teaching of cardiopulmonary resuscitation to nursing students. They found that using simulations and serious games in education could lead to a significant increase in the mean score of students’ knowledge and the skills of students [29]. However the authors were concerned regarding the effectiveness of their training approaches in imparting knowledge about CPR, as evidenced by low scores (below 70%) on posttest knowledge questionnaires by participants from both groups. The issue raises questions about whether the knowledge questionnaire was too challenging or if the training methods failed to convey the necessary CPR concepts effectively. Therefore, they suggest that incorporating direct instruction, such as lectures, might improve understanding of CPR knowledge. In our research, it was observed that the average knowledge scores for students in both groups exceeded 70% (14 out of 20), demonstrating the beneficial impact of both educational approaches on enhancing student knowledge levels. However, when assessing skill scores, it was observed that only the intervention group’s average skill scores surpassed the 70% threshold, indicating a level of skill considered desirable. These scores were also significantly higher compared to those of the control group.

Several studies have reported the effectiveness of serious games in knowledge improvement [41–46]. Serious games positively influence learning by providing interactive experiences that increase focus and motivation. These games enhance critical thinking, problem-solving, and decision-making skills, fostering cognitive development. Active participation in SGs promotes practical application of knowledge, improving retention and real-world applicability. Additionally, SGs increase users’ sense of control, encouraging the application of learned content in real-life situations [47]. The game’s characteristics such as attractiveness, variety, interactive environment, easy access without time limit, repeatability, the sense of competition, use of multimedia profiles, random questions, sharing of results, and invitation to play through social networks were considered as effective factors [44].

The advantages of serious gaming have also been applied to enhance practical skills, too. Johnsen et al. (2018) used a serious game (containing two simulated courses for providing care for patients with chronic obstructive pulmonary disease at home and in the hospital) with the aim of teaching clinical reasoning and decision-making skills to nursing students. They found that both courses were educationally valuable and easy to use for students. But serious games were highly acceptable among nursing students [48]. Other studies reported the positive effect of the serious games on the nursing students’ knowledge and skills in resuscitation of infants, including ventilation and chest massage [49] and adults [50]. Researchers have also focused on the use of serious games as facilitators of education in large groups [51–53]. Serious games have been shown to enhance practical and procedural abilities among nursing students by offering them an immersive and secure setting for improving their clinical reasoning and decision-making capabilities [54]. Nursing educators are encouraged to use SGs to enhance cognitive skills and attention, improve judgment, foster time-efficient decision-making, facilitate safe decision practices, and promote decision exploration [55]. In our research, students in the intervention group demonstrated significantly better skills at eight stations compared to the control group. Yet, when it came to evaluating the burn extent (station 9) and burn depth (station 10), the practical skills of both groups showed no significant differences. Given the critical role that understanding the extent and depth of burns plays in foundational training for subsequent topics, such as fluid resuscitation calculations, these topics were emphasized through repeated discussion and review, with numerous practical examples in the control group. Consequently, students in the control group could achieve comparable skills scores to those in the intervention group on stations 9 and 10. The significant differences observed in other variables may be attributed to the repeated exposure to educational content facilitated by the BAM game. In contrast, the control group received the information only once during the traditional lecture. Additionally, the gamification elements like competition, points, medals, encouraging emoji, and background music likely improved student engagement with the potentially disturbing content of the assessments of burn victims. These elements are typically absent in the standard classroom setting.

However, there are some conflicting findings. Dankbaar et al. (2017) addressed the effect of serious game on students’ knowledge of the principles of supporting patient safety and showed that although the serious game could improve students’ knowledge of patient safety, it had no effect on students’ practice regarding patient safety, so it was not different from traditional methods [56]. Tubelo et al. (2019) also showed no difference in improving students’ knowledge of primary health care between serious games and classroom teaching [57]. One possible reason for this contradiction is the difference in the methods and content used in these studies to train and assess students. For example, they focused on patient safety and primary health care, which had no unpleasant, stressful, threatening, and heartbreaking contents for students, so they could learn such contents by lecture method, while training burn patients or similar cases included many heartbreaking images and unpleasant scenes, and students were reluctant to frequently review and view these scenes.

### Strengths and Limitations

Some educational processes such as the assessment of burn patients are very stressful for students because they require more attention and concentration and any mistakes can threaten the patient’s life. The students of the intervention group in our study could experience better learning in a safe environment compared to the control group. We found no study that has compared serious game-based learning and traditional teaching methods in assessing burn patients.

One limitation of this study was that BAM is designed for assessing the most common types of burns in patients referred to our burn center, which may not be applicable to other departments or countries. Recruiting nursing students from only one university may be considered as another limitation, because it limits the generalizability of the findings. Small sample size was the third limitation, due to the small number of qualified students. On the other hand, this study was principally designed for a basic evaluation of our game and it will be developed during future phases.

## Conclusion

Nursing students’ lack of knowledge and weakness in clinical skills are one of the challenges of the educational system, which can have a negative impact on the prognosis of burn victims and the quality of life of those who recover from these injuries. If students do not acquire enough knowledge and skills to assess patients, they will be unable to correctly assess their problems and complications in the future. The serious game gives students the opportunity to access educational content without time limitation and experience unpleasant burn scenes in a game-like environment, making this method an effective and efficient educational method. In addition, students are eager to review the content due to the attractive environment of the game and try to correct their mistakes due to the competitive nature of the serious game. Therefore, we hope that this new educational method will be included in the lesson plan and educational curriculum of the nursing students due to its advantages over traditional methods.

This research has the potential to equip nursing managers and educators with new educational methods for enhancing students’ knowledge and assessment skills in caring for burn patients. Although, this research informs the development of improved clinical skills training, bridging the gap between theory and practice, a prevalent issue in the health education systems. By enhancing students’ knowledge and assessment skills, we can potentially reduce post-burn complications, lowering patient costs. Additionally, improved core assessments can minimize organ damage from acute complications, ultimately improving patient satisfaction and well-being.

### Electronic supplementary material

Below is the link to the electronic supplementary material.

Supplementary Material 1

## Data Availability

The datasets used and/or analysed during the current study are available from the corresponding author on reasonable request.

## Abbreviations

Burn assessment mission game: BAM game
GPA: Grade Point Average
T: Independent t-test
F: Fisher’s exact test
MW: Mann-Whitney test
