# Supplementary material for: Comparison of the effects of burn assessment mission game with feedback lecture on nursing students’ knowledge and skills in the burn patients’ assessment: a randomized clinical trial
Source: BMC Med Inform Decis Mak. 2024 Jun 5;24:157. doi: 10.1186/s12911-024-02558-4 (PMC11154992; doi:10.1186/s12911-024-02558-4)
Supplement: Supplementary file 1 — Supplementary Material 1 [file 12911_2024_2558_MOESM1_ESM.docx]

**Supplements:**

**Table S1: Frequency and percentage distribution of the skill scores in 10 station across intervention and control** **groups**

|  | Serious game | | | | | Feedback lecture | | | | |
| --- | --- | --- | --- | --- | --- | --- | --- | --- | --- | --- |
| Skills scores → | 1 | 2 | 3 | 4 | 5 | 1 | 2 | 3 | 4 | 5 |
| Skills score at station1 | 0 (0.0%) | 2 (4.8%) | 3 (7.1%) | 11 (26.2%) | 5 (11.9%) | 0 (0.0%) | 9 (21.4%) | 4 (9.5%) | 6 (14.3%) | 2 (4.8%) |
| Skills score at station2 | 0 (0.0%) | 0 (0.0%) | 5 (11.9%) | 9 (21.4%) | 7 (16.7%) | 0 (0.0%) | 9 (21.4%) | 8 (19.0%) | 3 (7.1%) | 1 (2.4%) |
| Skills score at station3 | 0 (0.0%) | 0 (0.0%) | 5 (11.9%) | 6 (14.3%) | 10 (23.8%) | 4 (9.5%) | 7 (16.7%) | 8 (19.0%) | 2 (4.8%) | 0 (0.0%) |
| Skills score at station4 | 0 (0.0%) | 2 (4.8%) | 5 (11.9%) | 12 (28.6%) | 2 (4.8%) | 3 (7.1%) | 6 (14.3%) | 7 (16.7%) | 4 (9.5%) | 1 (2.4%) |
| Skills score at station5 | 0 (0.0%) | 2 (4.8%) | 6 (14.3%) | 3 (7.1%) | 10 (23.8%) | 3 (7.1%) | 6 (14.3%) | 4 (9.5%) | 6 (14.3%) | 2 (4.8%) |
| Skills score at station6 | 0 (0.0%) | 0 (0.0%) | 2 (4.8%) | 10 (23.8%) | 9 (21.4%) | 3 (7.1%) | 8 (19.0%) | 1 (2.4%) | 6 (14.3%) | 3 (7.1%) |
| Skills score at station7 | 0 (0.0%) | 0 (0.0%) | 0 (0.0%) | 9 (21.4%) | 12 (28.6%) | 1 (2.4%) | 7 (16.7%) | 7 (16.7%) | 3 (7.1%) | 3 (7.1%) |
| Skills score at station8 | 0 (0.0%) | 0 (0.0%) | 0 (0.0%) | 6 (14.3%) | 15 (35.7%) | 2 (4.8%) | 6 (14.3%) | 3 (7.1%) | 4 (9.5%) | 6 (14.3%) |
| Skills score at station9 | 0 (0.0%) | 0 (0.0%) | 7 (16.7%) | 5 (11.9%) | 9 (21.4%) | 1 (2.4%) | 4 (9.5%) | 7 (16.7%) | 1 (2.4%) | 8 (19.0%) |
| Skills score at station10 | 0 (0.0%) | 7 (16.7%) | 0 (0.0%) | 12 (28.6%) | 2 (4.8%) | 4 (9.5%) | 6 (14.3%) | 1 (2.4%) | 8 (19.0%) | 2 (4.8%) |

Data are expressed as n (%)
